# Socioeconomic Differences in and Predictors of Home-Based Palliative Care Health Service Use in Ontario, Canada

**DOI:** 10.3390/ijerph14070802

**Published:** 2017-07-18

**Authors:** Jiaoli Cai, Denise N. Guerriere, Hongzhong Zhao, Peter C. Coyte

**Affiliations:** 1 School of Economics, Wuhan University of Technology, 122 Luoshi Road, Wuhan 430070, Hubei, China; jiaoli.cai@mail.utoronto.ca (J.C.); zhaohz22@163.com (H.Z.); 2 Institute of Health Policy, Management and Evaluation, University of Toronto, Health Sciences Building, 155 College Street, Suite 425, Toronto, ON M5T 3M6, Canada; denise.guerriere@utoronto.ca; 3 Canadian Centre for Health Economics, 155 College Street, Toronto, ON M5T 3M6, Canada

**Keywords:** Canada, home-based care, socioeconomic difference, health service use

## Abstract

The use of health services may vary across people with different socioeconomic statuses, and may be determined by many factors. The purposes of this study were (i) to examine the socioeconomic differences in the propensity and intensity of use for three main home-based health services, that is, home-based palliative care physician visits, nurse visits and personal support worker (PSW) hours; and (ii) to explore the determinants of the use of home-based palliative care services. A prospective cohort study was employed. A total of 181 caregivers were interviewed biweekly over the course of the palliative care trajectory, yielding a total of 994 interviews. The propensity and intensity of health service use were examined using logistic regression and negative binomial regression, respectively. The results demonstrated that both the propensity and intensity of home-based nurse and PSW visits fell with socioeconomic status. The use of home-based palliative care services was not concentrated in high socioeconomic status groups. The common predictors of health service use in the three service categories were patient age, the Palliative Performance Scale (PPS) score and place of death. These findings may assist health service planners in the appropriate allocation of resources and service packages to meet the complex needs of palliative care populations.

## 1. Introduction

Home-based palliative care programs provide services through community care teams consisting of physicians, nurses, personal support workers (PSW), and case managers [1]. These programs may assist patients to stay in their own home during the final stage of life. In Ontario, palliative home-based services based on need are currently funded by the publicly-funded health care system. However, patients and their families may also pay for additional services that are not covered by the public health care system. Canada has a universal, publicly-funded health insurance system with the aim to ensure that all residents have access to necessary health care services on the basis of need rather than financial status [2,3]. Little is known about whether patients with higher socioeconomic status can access more palliative home-based services in the presence of a publicly funded health system. 

Various aspects of home-based palliative care have been assessed in previous studies, such as health care costs [4,5,6,7,8,9], family caregiver burden [10,11,12,13], place of death [14,15], and the use of palliative care service [16,17,18,19]. However, there exists a paucity of research examining the socioeconomic differences in home-based palliative care health service use. Outside of the palliative care literature, some evidence indicates that people with higher financial status live longer and are in better health than those who are economically challenged [20,21]. Burge et al. [22] studied the relationship between home visits by family physicians (FP) and patient income in Nova Scotia (a Canadian Maritime province), and found that FP home visits were not associated with neighborhood income in a large metropolitan region of Halifax Regional Municipality (HRM). However, among patients living outside the HRM, those living in the higher income neighborhoods were more likely to receive a FP visit after adjusting for all other predictors. Although there exist studies that analyzed the relationship between socioeconomic condition and the use of health care resources in a general health care setting, few studies have examined this association in home-based palliative care. 

The purpose of this study was to examine whether or not there were socioeconomic differences in the propensity and intensity of health service use for the three main home-based services (physician visits, nurse visits and personal support worker (PSW) hours) over the course of the palliative care trajectory. Propensity was defined as the probability of receiving at least one service; and intensity referred to the number of health services used [23]. In addition, the determinants of the use of these home-based palliative care services were explored.

## 2. Methods 

### 2.1. Design

A prospective cohort sub-study was designed to capture the socioeconomic differences in health care service use among home-based palliative care patients. The data set was derived from a larger sample described elsewhere [24,25,26,27,28]. Family caregivers were recruited from a home-based palliative care program in Toronto, Canada: The Temmy Latner Centre for Palliative Care (TLCPC) at Mount Sinai Hospital. The TLCPC offers community- and team-based multidisciplinary palliative care (24 h a day, 7 days a week) to patients at home. Participants were eligible if they were (i) primary caregivers (family or friends) of patients who were diagnosed with a malignant neoplasm; (ii) fluent in English; and (iii) 18 years of age or older. Eligible caregivers were identified and contacted by palliative care program staff by telephone. Names and contact information of eligible caregivers who expressed interest were forwarded to the researcher, who then contacted the caregivers to provide additional study information and facilitate the consent and data collection process. Written consent was completed and mailed to caregivers, who then returned the consent form to the research team.

Primary caregivers were interviewed by telephone every two weeks from the time of study enrollment, which was just after the patient entered palliative care, from 1 July 2010 to 28 August 2012, until the death of the patient. Interviews lasted approximately 10–15 min. Caregivers were interviewed rather than patients because it would be difficult for patients to participate as their health status declined over the palliative care trajectory. A two-week period for data collection was selected because this was short enough to minimize recall bias, but not so short that it overburdened the family caregivers with too frequent interviews. This study was approved by both the University of Toronto and Mount Sinai Hospital ethics review boards.

The selection of potential predictors of health service use was based on the Andersen and Newman’s Behavioral Model of Health Service Utilization [29] and previous studies assessing predictors [25,26,27,28,30,31,32,33]. Andersen and Newman’s Behavioral Model considers individual health service utilization to be a function of three factors: predisposing factors; enabling factors, and; needs-based factors. Predisposing factors are individual characteristics which exist prior to the onset of specific episodes of illness. Such characteristics include demographic, social structural, and attitudinal-belief variables. A condition which permits a family to act on a value or satisfy a need regarding health service use is defined as enabling. Enabling conditions can be measured by family resources such as income and level of health insurance coverage. Needs-based factors represent the most immediate cause of health service use, and include symptoms that the individual experiences in a given time period, self-rated health status, and so forth. Thus, the potential predictors in our study comprised: predisposing factors (patient’s and caregiver’s age; sex and patient’s socio-demographic characteristics); enabling factors (socioeconomic characteristics), and; needs-based factors (the patient’s functional status).

### 2.2. Data Sources

Patient and caregiver demographic information was collected during the first interview and from the regional database by using the patient’s unique health card number. This information included the patient’s age, sex, marital status, deprivation score [34], education level, comorbidity score, and the caregiver’s age, sex, employment status, relationship with patient and caregiver burden. Place of death was also recorded in the regional database. In each interview, participants were asked to complete three questionnaires: The Ambulatory and Home Care Record (AHCR) [24]; the Palliative Performance Scale (PPS) [35], and; the Caregiver Burden Scale in End-of-Life Care (CBS-EOLC) [36].

The AHCR (© Coyte & Guerriere 1998) was used to collect health service use (the number of nurse and physician visits and PSW hours of care) in each interview [24,30]. The AHCR is a tool designed to measure resource use [24] and has been used in a variety of clinical settings [6,25,26,28] and has been demonstrated to yield valid and reliable data [37]; a moderate to high level of agreement was found between participants’ responses and administrative data (kappa: 0.41–1.00) [37]. The number of publicly funded home-based physician and nurse visits, and PSW hours were recorded in the AHCR. Family caregivers were asked to recall whether, over the previous two weeks, the patient received any of these services or not, and to report the frequency of service use if the patient received the specific service.

In each interview, the PPS was used to collect data on the functional and cognitive status of patients. The PPS score can range from 0 to 100, with incremental changes of 10. A score of 0 indicates death and a score of 100 indicates that the patient is ambulatory and healthy [35]. Thus, a higher PPS score indicates better functional status. Reliability testing of the PPS reported intra-class correlation coefficients ranging from 0.93 to 0.96 [38,39].

Caregiver burden was measured using the CBS-EOLC, a 16-item questionnaire using a 4-point Likert scale [36]. The total score can range from 16 to 64. A higher score indicates a higher burden. Assessment of the psychometric properties demonstrated appropriate reliability of the scale (Cronbach’s alpha = 0.95) and good levels of convergent validity with fatigue (r = 0.69) and depression (r = 0.54) [36].

In addition, socioeconomic status was measured using the Carstairs deprivation score and the education level of the patient [40]. Canadian census data linked to the patient’s postal code were used to compute a modified Carstairs deprivation score [34]. Specifically, the deprivation score was calculated based on each region’s standardized measures: (i) percentage of blue-collar workers among men (social class); (ii) the percentage of unemployment rate among men, and; (iii) the percentage of those living in the households which were below the low-income cutoff. A higher deprivation score indicates lower socioeconomic status. Educational status was collected using three categories (high school or less, any university and post-graduate).

### 2.3. Statistical Analysis

In order to assess whether socioeconomic differences existed in health service use, Pearson’s χ^2^ test was implemented to examine whether statistically significant differences existed in the propensity of health service use across different socioeconomic status. One-way analysis of variance (ANOVA) was used to test whether statistically significant socioeconomic differences existed in the intensity of health service use. Logistic regression was employed to analyze the propensity of health service use; while negative binomial regression was utilized to analyze the intensity of health service use. Compared to the models with forward stepwise regression or the models without stepwise selection in regression, we found that the models with backward stepwise regression were better according to the Pseudo R^2^ statistics. This is due to the fact that we got higher Pseudo R^2^ values in the models with backward stepwise regression, which indicated that the covariates controlled for can better predict the dependent variable. Thus, we conducted backward stepwise regression on our data. Through backward stepwise regression analysis, with a significance level of 0.10 to stay in the model, the final multivariate model including all significant variables was derived. All analyses were performed using Stata (Stata version 13.0 for Mac, StataCorp LP, College Station, TX, USA).

## 3. Results

Over the data collection period (from 1 July 2010 to 28 August 2012), 805 caregivers of patients were identified as being eligible. Of these potential participants, 88 were not approached as the attending physicians noted that the caregiver was in extreme stress. Of the remaining 717 potential caregiver-participants, 552 (77.0%) caregivers were contacted by the research officer; of the caregivers who were contacted, 367 (66.5%) agreed to receive further information, and of these 341 (92.9%) caregivers agreed to participate in the study. Of these participants, 14 caregivers were ineligible at the first interview because the patient had moved (n = 1), was hospitalized (n = 4), or the patient had died (n = 9). Additionally, 18 caregivers were excluded because of unknown date of death, and 128 caregivers were excluded from the analysis due to missing data for some potential predictors, such as education level. Though there were some missing data for some variables, based on the Little’s chi-squared test [41] and a series of independent t-tests [42], we found that the data that were missing were missing completely at random. So there was no difference between the missing cases and the complete cases. Consequently, the findings were based on a group of 181 participants. The participants yielded a total of 994 interviews. The mean number of interviews per caregiver was 5.5, with a minimum of one interview and a maximum of 37 interviews per caregiver.

### 3.1. Patient and Caregiver Characteristics

Table 1 presents the descriptive statistics for the study sample. The mean patient age was 71.9 years, with a nearly equal split between women (54.14%) and men (45.86%). More than forty percent of the patients reported having a high school diploma or less (43.09%). Most of the patients lived with others (78.45%) and 57.46% were married. The mean caregiver age was 58.5 years, and 65.75% were women. Thirty-four percent of the caregivers were retired, and 45.3% were the patient’s spouse.

### 3.2. Socioeconomic Difference in Health Service Use 

The use of the three categories of home-based palliative care services is displayed in Table 2. For all of the 994 interviews, home-based nurse visits were the most common service category reported (85.31%), followed by home-based palliative care physician visits (60.46%). Table 3 presents the differences in health service use by socioeconomic level. Based on the result of Pearson’s χ^2^ test, we found that significant differences existed in the propensity of service use, particularly for home-based nurse visits and home-based PSW visits across the socioeconomic levels. Patients with a higher deprivation score (i.e., with a lower socioeconomic status), were more likely to receive at least one home-based nurse visit or PSW visit than those who had lower deprivation scores. Based on the result of one-way ANOVA, we found that there was also a statistically significant and negative association between socioeconomic status and the intensity of health service use. For example, the average number of home-based nurse visits was 6.28 for those in the top quartile of the deprivation score (i.e., those with the lowest socioeconomic status), compared to 4.42 for those in the bottom quartile and with the highest socioeconomic status (*p* < 0.001). 

### 3.3. Predictors of Health Service Use

Table 4 and Table 5 present the results of the final logistic regression and the negative binomial regression using backward stepwise regression analysis, respectively. Socioeconomic status was a significant predictor of the propensity and the intensity of home-based nurse and PSW visits, even after controlling for health status. The higher the deprivation score (lower socioeconomic status), the higher the propensity and the intensity of home-based nurse and PSW visits. Patients with higher levels of educational attainment were less likely to receive at least one home-based PSW visit. Older patients had a higher propensity and intensity of home-based PSW visits, but a lower propensity and intensity of both home-based physician visits and nurse visits. Male patients were less likely to receive at least one home-based PSW visit, and had a lower intensity of use for the three home-based services. Patients who had never been married were less likely to receive at least one home-based nurse visit, but were more likely to receive at least one home-based PSW visit. Divorced, separated or widowed patients had a 64% lower likelihood of having at least one home-based nurse visit and had a lower intensity of home-based PSW visits. Patients’ comorbidity scores were positively associated with the propensity and intensity of PSW visits. Patients with higher PPS scores had a higher propensity and intensity of home-based physician and PSW visits, but were less likely to receive home-based nurse visits. Patients who lived alone had a 54% and 56% lower likelihood of receiving at least one home-based nurse or PSW visit, respectively. The propensity and intensity of home-based physician and nurse visits increased with proximity to death. Patients who died at home had a higher propensity and intensity to use all three of the health services. Patients who had male caregivers had a 67% and 29% lower likelihood of having at least one home-based nurse and PSW visit, respectively, and had a lower intensity of home-based PSW visits. Patients who were cared for by spousal caregivers had a 77% lower likelihood to receive home-based nurse visits, and had a 14% and 29% lower intensity of home-based physician and nurse visits, respectively. Caregiver burden increased the likelihood that the patient would have a home-based physician visit.

## 4. Discussion

This is the first study to examine socioeconomic differences in the propensity and intensity of home-based health service use for three main palliative care services. The socioeconomic status of those dying of cancer was negatively associated with the propensity and intensity of home-based nurse and PSW visits after controlling for health status, but was not associated with the propensity and intensity of home-based palliative care physician visits. Those with higher socioeconomic status had both a lower propensity and intensity of home-based nurse visits and PSW hours. This finding may be due to the greater opportunity of their caregivers to take time away from work. Indeed, we found that the average number of unpaid caregiving hours provided for those who had the highest socioeconomic status and the lowest socioeconomic status was 6.18 and 2.66, respectively, over each interview period. This finding may be also because that patients with higher socioeconomic status tended to use other additional services which were not supplied through the publicly funded system. Our finding was consistent with the finding by Grande et al., who found that patients with a higher deprivation score were more likely to access palliative care services in the United Kingdom [17]; however, the service categories that they assessed were different from ours and were community nurse specialist advice, nursing and inpatient hospice care, and they did not look at service intensity. Aylin et al. [43] reported that individuals with the greatest income received the fewest home visits by general practitioners in the United Kingdom. The authors explained that the prevalence of home visits was common among those with lower income because of a number of factors, such as increased morbidity and less access to a car. However, their result was not directly comparable to our result, as they focused on general practitioners. Another older study conducted in Canada showed that, due to the implementation of universal health care, the use of physician services increased more for those in low-income compared to those in high-income groups; however, their result was also not directly comparable to ours, as they focused on general health care service, instead of palliative care service [44].

The second purpose of our study was to explore the predictors of home-based palliative care service use. We found that patient age, sex, marital status, living arrangement, comorbidity score, PPS level and place of death were important predictors of health service use. Older patients had a lower propensity and intensity of home-based physician and nurse visits than younger patients. Similar results were also reported by a previous Canadian study [25]. However, in our study older patients were more likely to receive at least one home-based PSW visit, and had a higher intensity of home-based PSW visits than younger patients. Patients of different ages receiving different services may be due to patient or caregiver preferences. One study found that caregiver age was an important predictor of palliative home care use [17]. Caregivers of different ages may express a desire for a different service mixture.

Male patients had a lower propensity to receive home-based PSW care, and a lower intensity to use the three services. This finding may be attributed to greater access to unpaid caregivers. As we found in the survey, the average number of unpaid caregiving hours received by men for each interview period was slightly greater at 3.77 than that received by women at 3.49. One Canadian study found that the costs of home-based palliative care service varied by gender. The costs were $17,814.15 for each female patient and $16,395.29 for men with comparable lengths of stay in the program at 145 days on average [9]. 

Patients who had never been married were less likely to receive home-based nurse visits compared to those who were married. Patients who lived alone were less likely to receive home-based nurse and PSW services. This may be because of the absence of individuals to advocate on their behalf and to arrange these services [45,46]. Another study also found patients who were married and lived with others were more likely to receive care from community specialist palliative care (CSPC) nurses [16]. The authors explained that if the patient had a caregiver, they were more likely to reach out for services. In addition, patients who were never married were more likely to receive home-based PSW service because they probably have access to fewer unpaid caregiving hours. As we found in the survey, the average number of unpaid caregiving hours received by patients who were never married was only 2.29 h over the interview period; while it was 4.23 h for patients who were married.

In our study, comorbidity score was a positive predictor for both the propensity and intensity to receive home-based PSW visits. One study found the comorbidity index was strongly associated with death [47]. Patients with a lower PPS score (i.e., lower functional status), had a higher propensity and intensity to receive home-based nurse visits. This finding is expected; as patients’ health deteriorates, more health care services are required. This was consistent with the finding by Maltoni et al. [48], who found health care costs increased as the Karnofsky Performance Status (KPS) (an interchangeable tool with the PPS) decreased. In contrast, in our study, patients with a higher PPS score were more likely to receive and had a higher intensity of home-based physician and PSW visits, which was unexpected. This may be because we used three variables (PPS score, comorbidity score and caregiver burden) jointly to represent the health status of the patient. Proximity to death increased the propensity and intensity of home-based physician and nurse visits. It is expected that patients may need more health services because of the sharp decline in their health state when patients are close to death. Other studies have demonstrated an increase in the cost of health services as death approaches [8,26,30,49]. Patients who died at home had both a higher propensity and intensity to receive each of the three home-based health services. This may be because the increased provision of services to these patients gives them a greater ability to die at home.

Caregivers characteristics were also correlated with health service use. Patients who were cared for by male caregivers had a lower propensity to receive a home-based nurse or PSW visit, and a lower intensity of home-based PSW visits. This may be because female caregivers lobbied more for those services compared to male caregivers. Patients cared for by spousal caregivers were less likely to receive a home-based nurse visit, and had a lower intensity of home-based nurse and physician visits. A previous Canadian study reported similar results and the authors suggested a possible explanation that a portion of nurses’ work could be fulfilled by spousal caregivers [19]. Caregiver burden was an important predictor for the propensity to receive a home-based physician visit. An increase in caregiver burden may be symptomatic of a decline in the patient's health status [27], and hence, the need for home-based physician visits. 

There were some limitations of this study. First, this study was based on one home-based palliative care program in Toronto. The findings here may not necessarily generalize to other palliative care settings. However, the population serviced by this palliative program is diverse in clinical, demographic and ethnic background, which may help improve generalizability. Second, because some of the data were acquired from telephone interviews with caregivers, there may be a potential for recall bias and social desirability bias. However, because the psychometric properties of the study instruments have met acceptable standards, we believe this bias, if present, may be small. Third, the sample size was relatively limited. However, small sample studies are common in palliative care studies, and the model we used in this study was able to consider most of the key variables needed for the analysis.

## 5. Conclusions

This study examined the association between socioeconomic status and health service use, and explored the predictors for the use of various categories of home-based palliative care. We advance three main findings. First, the use of home-based palliative care services was not concentrated in high socioeconomic status groups after controlling for health status and other potential factors, which implies that higher socioeconomic status does not result in greater access to these services. Second, fully consistent predictors for home-based health service use across the three service categories did not exist, thereby implying that utilization for each service category is unique from that for other services. Third, the determinants for each of the two components to utilization, namely propensity and intensity of use, were also distinct.

Socioeconomic status was not the main factor to influence health service utilization in the presence of a publicly-funded health insurance system in Canada. The main driver of home-based palliative care utilization was the patient’s need for care as indicated by the importance of the PPS, the comorbidity score and place of death. Additionally, utilization was also driven by both predisposing and enabling factors. Understanding the predictors for the use of home-based palliative care can assist service planners in the appropriate allocation of resources and service packaging to meet the complex needs of palliative care patients. As more and more patients receive their palliative care at home, families who require financial and psychosocial support to actualize this preference may benefit from studies that explore the predictors of home-based health service use.

## Figures and Tables

**Table 1:** Descriptive statistic for the study sample (n = 181).

Variables	Category	Number	%
*Patient characteristics*			
Age (year): mean 71.9, median 73, SD ^a^ 13.23	≤61	39	21.55
	62–72	49	27.07
	73–82	45	24.86
	≥83	48	26.52
Sex	Female	98	54.14
	Male	83	45.86
Marital status	Married	104	57.46
	Divorced, separated, or widowed	70	38.67
	Never married	7	3.87
Socioeconomic status			
Deprivation score: mean 0.72, median 0.68, SD 0.42	Quartile 1 (0.06–0.45)	49	27.07
	Quartile 2 (0.46–0.68)	42	23.21
	Quartile 3 (0.70–1.04)	45	24.86
	Quartile 4 (1.07–2.23)	45	24.86
Education level	High school or less	78	43.09
	Any university	76	41.99
	Post-graduate	27	14.92
Comorbidity score: mean 6.87, median 7, SD 1.16	6–7	142	78.45
	8–9	33	18.23
	10–12	6	3.32
Baseline PPS ^b^ level: mean 28, median 28, SD 7.67	8–30	116	64.09
	31–50	65	35.91
Living arrangement	Live with others	142	78.45
	Live alone	39	21.55
Death place	Other place	90	49.72
	Home	91	50.28
*Caregiver characteristics*			
Age (year): mean 58.5, median 59, SD 13.27	≤45	28	15.47
	46–69	119	65.75
	≥70	34	18.78
Sex	Female	119	65.75
	Male	62	34.25
Employment status	Others	120	66.3
	Retired	61	33.7
Relationship with patient	Others	99	54.7
	Spouse	82	45.3
Baseline caregiver burden: mean 27, median 26, SD 7.60	16–30	133	73.48
	31–57	48	26.52

^a^ Standard deviation; ^b^ Palliative Performance Scale.

**Table 2:** Propensity and intensity of service use by service category (n = 994).

Dependent Variable		Physician	Nurse	PSW ^a^
Propensity	Yes	601	848	560
	No	393	146	434
	Proportion of use (%)	60.46	85.31	56.34
Intensity	Mean ^b^	0.89	5.02	1.20

^a^ Personal support worker. ^b^ Average unconditional number of visits over the palliative care trajectory.

**Table 3:** The distribution of health service use across socioeconomic status (n = 994).

Variables	Category	Physician		Nurse		PSW ^a^	
		Propensity	Intensity	Propensity	Intensity	Propensity	Intensity
		n (%)	Mean	n (%)	Mean	n (%)	Mean
Deprivation score	Quartile 1 (0.06–0.38)	146 (57.94)	0.74	209 (82.94)	4.42	147 (58.33)	0.89
	Quartile 2 (0.44–0.70)	163 (66.26)	1.08	207 (84.15)	4.30	127 (51.63)	1.17
	Quartile 3 (0.74–1.00)	162 (63.28)	0.93	216 (84.38)	5.11	152 (59.38)	1.22
	Quartile 4 (1.04–2.23)	130 (54.17)	0.83	216 (90)	6.28	134 (55.83)	1.51
*p* value		0.030	0.006	<0.001	<0.001	<0.001	<0.001
Education level	High school or less	246 (60)	0.82	332 (80.98)	4.71	261 (63.66)	1.29
	Any university	233 (60.21)	0.93	339 (87.6)	5.72	230 (59.43)	1.25
	Post-graduate	122 (61.93)	0.98	177 (89.85)	4.26	69 (35.03)	0.90
*p* value		0.894	0.020	0.004	<0.001	<0.001	<0.001

^a^ Personal support worker.

**Table 4:** Predictors of the propensity to use the three service (Logistic regression).

Variables	Category	Physician	Nurse	PSW ^c^
		OR ^a^ (95% CI ^b^)	OR (95% CI)	OR (95% CI)
*Patient*				
Age		0.98 *** (0.97–0.99)	0.97 *** (0.95–0.99)	1.04 *** (1.02–1.05)
Sex	Female			1.00
	Male			0.28 *** (0.19–0.40)
Marital status	Married		1.00	1.00
	Divorced, separated, or widowed		0.36 ** (0.13–0.99)	1.40 (0.90–2.19)
	Never married		0.05 *** (0.01–0.16)	2.59 ** (1.10–6.09)
Deprivation score			2.11 *** (1.21–3.66)	1.53 ** (1.09–2.14)
Education level	High school or less			1.00
	Any university			1.11 (0.79–1.57)
	Post-graduate			0.57 ** (0.37–0.88)
Comorbidity score				1.40 *** (1.23–1.60)
PPS ^d^ level		1.02 ** (1.00–1.04)	0.95 *** (0.93–0.98)	1.03 *** (1.01–1.04)
Living arrangement	Live with others		1.00	1.00
	Live alone		0.46 ** (0.24–0.88)	0.44 *** (2.30–0.71)
Time to death		0.99 *** (0.99–1.00)	0.99 *** (0.99–1.00)	
Death place	Other place	1.00	1.00	1.00
	Home	1.30 * (0.99–1.70)	3.44 *** (2.13–5.56)	1.79 *** (1.31–2.44)
*Caregiver*				
Sex	Female		1.00	1.00
	Male		0.33 *** (0.21–0.51)	0.71 * (0.50–1.04)
Relationship with patient	Others		1.00	
	Spouse		0.23 *** (0.09–0.59)	
Caregiver burden		1.01 * (1.00–1.03)		

^a^ Odds ratio; ^b^ Confidence interval; ^c^ Personal support worker; ^d^ Palliative Performance Scale; *** *p* < 0.01, ** *p* < 0.05, * *p <* 0.1.

**Table 5:** Predictors of the intensity to use the three service (Negative binomial regression).

Variables	Category	Physician	Nurse	PSW ^c^
		IRR ^a^ (95% CI ^b^)	IRR (95% CI)	IRR (95% CI)
*Patient*				
Age		0.99 *** (0.98–0.99)	0.99 *** (0.99–1.00)	1.02 *** (1.01–1.02)
Sex	Female	1.00	1.00	1.00
	Male	0.86 ** (0.75–0.99)	0.65 *** (0.57–0.75)	0.52 *** (0.43–0.64)
Marital status	Married			1.00
	Divorced, separated, or widowed			0.76 *** (0.63–0.92)
	Never married			0.90 (0.55–1.46)
Deprivation score			1.40 *** (1.22–1.60)	1.18 ** (1.01–1.37)
Comorbidity score				1.17 *** (1.09–1.26)
PPS ^d^ level		1.02 *** (1.01–1.03)	0.99 *** (0.99–1.00)	1.04 *** (1.04–1.05)
Time to death		0.99 *** (0.99–1.00)	1.00 *** (0.99–1.00)	
Death place	Other place	1.00	1.00	1.00
	Home	1.17 ** (1.02–1.35)	1.48 *** (1.29–1.70)	1.67 *** (1.41–1.97)
*Caregiver*				
Sex	Female			1.00
	Male			0.62 *** (0.50–0.76)
Relationship with patient	Others	1.00	1.00	
	Spouse	0.86 ** (0.74–1.00)	0.71 *** (0.62–0.82)	

^a^ Incidence-rate ratio; ^b^ Confidence interval; ^c^ Personal support worker; ^d^ Palliative Performance Scale; *** *p <* 0.01, ** *p <* 0.05, * *p <* 0.1.
